# Probable and confirmed sarcopenia are still better predictors of disability than sarcopenic obesity following ESPEN/EASO consensus steps

**DOI:** 10.1186/s12877-025-05897-7

**Published:** 2025-04-15

**Authors:** Sibel Cavdar, Fatma Ozge Kayhan Kocak, Sumru Savas

**Affiliations:** 1 Department of Internal Medicine, Division of Geriatrics, Ege University Hospital, Izmir, Turkey; 2 Department of Internal Medicine, Division of Geriatrics, Izmir City Hospital, Izmir, Turkey; 3 Department of Internal Medicine, Division of Geriatrics, University of Health Sciences Tepecik Education and Research Hospital, Izmir, Turkey

**Keywords:** Sarcopenia, Sarcopenic obesity, ESPEN/EASO consensus criteria, Functional dependency, Disability, EWGSOP2

## Abstract

**Background:**

Studies comparing different operational definitions of sarcopenia (S) and sarcopenic obesity (SO) defined according to the ‘’European Society for Clinical Nutrition and Metabolism and the European Association for the Study of Obesity’’ (ESPEN/EASO) criteria with functionality are scarce. Our aim is to investigate whether SO or S with different skeletal muscle mass (SMM) adjustments is better associated with functional disability.

**Methods:**

This retrospective study was carried out in older individuals **≥** 65 years of age in a geriatric outpatient clinic. Probable and confirmed sarcopenia were evaluated with the revised European Working Group on Sarcopenia in Older People (EWGSOP2) criteria, and SO with ESPEN/EASO consensus steps. For SMM component for both S and SO, different adjustments (weight, body mass index, and height square (W, BMI, H^2^ respectively)) were used. Functional disability was examined with activities of daily living (ADL), and instrumental ADL (IADL). Receiver operating characteristic (ROC) curves were drawn and area under ROC curve (AUC) were calculated to find which operational definition best predicts disability.

**Results:**

Data from 1477 older adults were screened. 408 participants (median age; 73 (65–101), 65% female) were included. Prevelance of SO was 6.9%. Probable sarcopenia, confirmed sarcopenia BMI-adjusted and confirmed sarcopenia W-adjusted were significantly associated with impaired IADL (*p* < 0.001), and showed fair accuracy for predicting IADL disability. Sarcopenic obesity did not show significant associations with ADL and IADL disability and didn’t predict ADL and IADL disability. Only confirmed sarcopenia by BMI predicted ADL disability with poor accuracy. Among operational definitions of sarcopenia, probable sarcopenia had the highest sensitivity (83.6%) and negative predictive value (NPV) (94.2%) for predicting IADL disability.

**Conclusion:**

We found that probable sarcopenia (with the highest sensitivity and NPV) and confirmed sarcopenia (BMI-adjusted with higher sensitivity and NPV than W-adjusted) were the most relevant for predicting IADL disability, but their diagnostic accuracy was limited. Confirmed sarcopenia by BMI predicted ADL disability with poor accuracy. Other operational definitions, including SO did not predict functional disability in our study. Future studies need to refine the definitions of SO and investigate its distinct impact on functional impairment compared to sarcopenia alone.

## Introduction

With the rapid growth of the older population globally, sarcopenic obesity (SO) is estimated to affect 100–200 million older people in nearly 3 decades [1]. SO is a high-risk geriatric syndrome which has the cumulative health risks of both phenotypes synergistically and it threatens the health and quality of life [2–4]. SO is identified as the coexistence of sarcopenia (S) and obesity (O) components. For sarcopenia component, there are several definitions, methodologies and thresholds according to different working groups [5–10]. In revised European Working Group on Sarcopenia in Older People (EWGSOP2) consensus, detection of low muscle strength is defined as probable sarcopenia, while the presence of low muscle strength and low muscle mass together is defined as confirmed sarcopenia [6]. Though the revised EWGSOP (EWGSOP2) consensus for the definition and diagnosis of sarcopenia has suggested cut-off points to provide harmonization among studies, the use of regional normative populations is also recommended when available as measurements such as gait speed and strength depend upon stature [6]. For the definiton of obesity, there are also several definitions such as body mass index (BMI), waist circumference, and fat mass with different thresholds [11–13]. These variations in definitions have led problems in comparing SO prevalences and the other results across studies [11, 14]. In addition, this situation continues to hinder the implementation of primary and secondary treatments and recommendations. European Society for Clinical Nutrition and Metabolism and the European Association for the Study of Obesity (ESPEN/EASO) published a standardized definition and diagnostic criteria as ESPEN/EASO consensus criteria in 2022 [14]. The authors of the consensus paper encouraged studies on functionality with this algorithm and also is indicated that future research should aim at defining the best cut points to be considered in research and clinical practice concerning SO.

It is known that there are independent negative effects of sarcopenia and obesity on physical functioning. Besides, older adults with SO may experience poorer functional outcomes and lower physical performance compared with those with sarcopenia or obesity alone [15–17]. Obesity and sarcopenia may synergistically reinforce each other, creating a vicious cycle of fat gain and muscle loss through reduced mobility, dependence and disability [18]. On the other hand there are studies with different results such as obesity may have a protective effect against the limitations of some functional measures and physical performance, advocating the protective effect of obesity in sarcopenic individuals [19–21].

To our knowledge after the ESPEN/EASO consensus criteria, the studies investigating the assosiations between SO and functionality have still conflicting results as before this consensus. While some of the studies justified SO was associated with disability and worse functional outcomes in different patient groups [22–25], some of the studies found SO was not associated with poor functional outcomes [26, 27], or with activities of daily living [28] in different situations. However studies comparing different operational definitions of S and SO by ESPEN/EASO with functionality are scarce [23].

Another inconsistent issue is about skeletal muscle mass (SMM) adjustments of S component of SO. In ESPEN/EASO consensus statement SMM adjustment by weight (W) is suggested [14], and in EWGSOP2 SMM adjustment by height square (H^2^) is suggested [6]. However, when the SMM is adjusted by BMI, low muscle mass showed better associations with ADL, IADL, frailty and risk of falls than the H^2^ or the weight-adjusted SMM [29–32]. Furthermore, BMI adjustments of SMM is encouraged in SO studies diagnosed using ESPEN and EASO on functional outcomes [29, 31].

For these reasons, we aimed to find which operational definition of S and SO with local thresholds with different adjustments (W, H^2^, and BMI) following ESPEN/EASO consensus steps is more associated with functional disability, since preserving functionality for as long as possible is one of our main goals for the older population.

## Materials and methods

### Study design and population

**Fig. 1:**
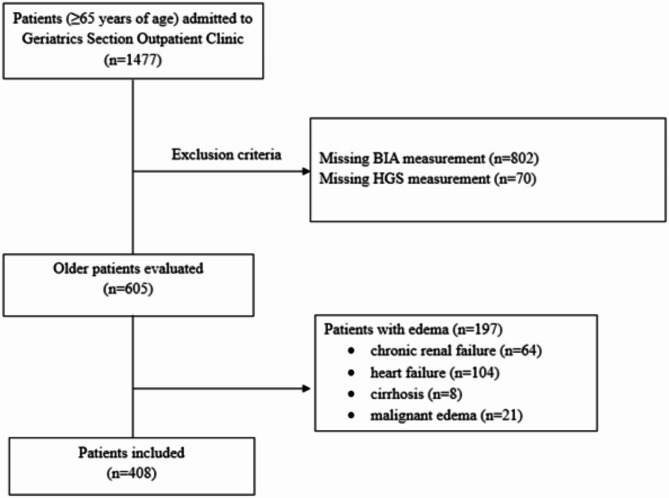
Exclusion and inclusion criteria of the study population

This study was carried out in older individuals **≥** 65 years of age applied to the outpatient clinic of Geriatric Medicine between July 2016 and March 2021. From 1477 participants, we excluded 802 participants with missing bioelectrical impedance analysis (BIA) measurements, 70 participants with missing handgrip strength (HGS) measurements and 197 patients with edema with different health problems such as chronic renal failure, heart failure, cirrhosis and malignant edema leading to a sample size of 408 participants (265 women). Exclusion and inclusion criteria are shown in Fig. 1. Socio-demographic data, BMI, BIA values, number of medications, as well as comorbidities, HGS values etc. were recorded for all patients. All patients’ data were retrieved retrospectively from hospital records.

### Screening for sarcopenic obesity

Body composition measurements were assessed with a multifrequency tetrapolar instrument (Tanita MC-780BIA) at 50 kHz. The definitions and diagnostic steps of the ESPEN and EASO consensus statement with local thresholds were used to identify patients with SO [14]. The ESPEN and EASO criteria consist of three steps: screening, diagnosing and grading of the severity of the SO. Total SMM and fat mass were calculated using the BIA equation developed by Janssen et al. [33] and Gallagher et al. respectively [34]. Body mass index was calculated by dividing weight in kilograms by the square of height in meters. The SMM was also adjusted for weight (SMM/W) and BMI (SMM/BMI). BMI greater than or equal to 30 (BMI ≥ 30 kg/m²) was defined as obesity for SO screening tool [14]. Depending on gender, ethnicity and examination method, the specific cut-off values for SO are modified. In our study, we used the local cut-off values shown in Table 1.

**Table 1:** The cut-off values for assessment of sarcopenic obesity according to sex

The criteria	Threshold value	
	Men	Women
Low handgrip strength [35], kg	35	20
Increased fat mass [13], %	27.3	40.7
Reduced muscle mass by BIA		
SMM/W, %* [36]	27.53	23.26
SMM/BMI* [36]	0.82	0.58

BIA: the bioelectrical impedance analysis, SMM: skeletal muscle mass, W: weight, BMI: body mass index

*SMM was calculated according to Janssen equation in the given reference [36]

### Screening for sarcopenia

Muscle strength was assessed by HGS measured by Takei T.K.K. 5401 digital dynamometer (Takei Scientific Instruments Co. Ltd, Tokyo, Japan) implementing a validated protocol [37]. Total SMM was calculated using the BIA equation developed by Janssen et al. [33] Sarcopenia was defined according to EWGSOP2 criteria [6]. Based on this algorithm probable sarcopenia was defined as the presence of low handgrip strength, and confirmed sarcopenia was defined as low muscle strength and low SMM. The EWGSOP2 consensus states that skeletal muscle mass should be adjusted for height square [6]. The local cut-off values in Table 2 were used for assessment of sarcopenia.

**Table 2:** The cut-off values for criteria of operational definition of sarcopenia according to sex

The criteria	Threshold value	
	Men	Women
Low handgrip strength [35], kg	35	20
Reduced muscle mass by BIA		
SMM/W, %* [36]	27.53	23.26
SMM/BMI* [36]	0.82	0.58
SMM/(H^2^)** [38], kg/m^2^	8.33	5.70

BIA: the bioelectrical impedance analysis, SMM: skeletal muscle mass, W: weight, BMI: body mass index, H^2^: height square

*SMM was calculated according to Janssen equation in the given reference [36]

** SMM was calculated according to Janssen equation in the given reference [38]

### Assessment of functionality

Functional status was assessed by KATZ ADLs [39], and Lawton-Brody IADLs [40]. The ADLs refer to the six activities of daily living (bathing, dressing, feeding, ambulation, toileting, continence) while the IADLs require more complex planning and thinking acts such as managing medications, paying bills, and using the telephone. The total scores for ADLs and IADLs scales were 6 and 8 points, respectively. The patients were evaluated as “disabled” by ADLs and IADLs scales, if scores were < 6, and < 8, respectively.

### Sample size estimation

The sample size of the study was calculated using G*Power 3.1.9.7. The study by Bahat et al. was used as a reference for the effect size to be used in the calculation [19]. Based on the stated prevalence of SO leading to impaired IADL, with a 0.05 type I error and 80% power, the minimum required sample size for the study was calculated as 345.

### Statistical analysis

Data analyses were performed using SPSS version 25.0 for Windows. *P* ≤ 0.05 was considered statistically significant. Data normality was obtained by Kolmogorov-Smirnov test. The T test and Mann-Whitney U test were used in the analysis of quantitative variables where available. The chi-squared (χ2) test and Fisher’s exact test were used for the comparison of categorical variables. Normally distributed quantitative variables, quantitative variables without normal distribution and qualitative variables were expressed by mean ± standard deviations, median (minimum-maximum) and frequency (percentages) respectively.

To evaluate the diagnostic performance of the different operational definitions of sarcopenia, the following parameters were calculated: sensitivity, specificity, positive predictive value (PPV), negative predictive value (NPV), as well as the area under the receiver operating characteristic (ROC) curve (AUC). We used the 2 × 2 cross-tabulation for calculating specificity, sensitivity, PPV, and NPV at the diagnostic accuracy of the different operational definitions of sarcopenia for functional disability [41]. Sensitivity and specificity were classified as good (> 80%), fair (50–80%), or poor (< 50%) [42]. The AUC is a measure of the overall diagnostic accuracy of a test. An AUC > 0.8 indicates good, 0.6–0.8 fair, and < 0.6 indicates poor diagnostics accuracy [43].

## Results

### Study population characteristics

The mean age was 73.5 (65–101) years in women, 75 (65–94) years in men. Females composed 65% of the study population. Individuals who had SO according to calculation adjusted by weight (6.9%) were the same patients according to calculation adjusted by BMI (6.9%). 237 (58.1%) participants had probable sarcopenia. 116 (28.4%) of all had confirmed sarcopenia and 28 (6.9%) had SO with both W and BMI adjustments. The descriptive statistics of study sample was shown in Table 3.

**Table 3:** Characteristics of the study sample

Age, years	73 (65–101)
Sex, female	265 (65%)
ADL score	6 (0–6)
IADL score	8 (0–8)
HGS, kg	23 (6–52)
BMI, kg/m^2^	28.2 (16.2–54.3)
SMM/W, %	25.6 (13.09–47.44)
SMM/BMI	0.6 (0.3–1.3)
SMM/H^2^, kg/m^2^	11.3 (5.5–21.9)
Fat mass, %	31.2 (5.8–51.3)
Impaired ADL	203 (49.8%)
Impaired IADL	61 (15%)
Sarcopenia parameters	
Low HGS	237 (58.1%)
Low SMM/W	149 (36.5%)
Low SMM/BMI	203 (49.8%)
Low SMM/H^2^	1 (0.2%)
Increased fat mass	72 (17.6%)
Different operational definitions	
Probable sarcopenia	237 (58.1%)
Confirmed sarcopenia/W	76 (18.6%)
Confirmed sarcopenia/BMI	116 (28.4%)
Confirmed sarcopenia/H^2^	1 (0.2%)
Sarcopenic obesity/W	28 (6.9%)
Sarcopenic obesity/BMI	28 (6.9%)
Obesity	149 (36.5%)

Continuous variables expressed as a median (minimum-maximum); categorical variables expressed as number (frequency). ADL: activities of daily living, IADL: instrumental activities of daily living, HGS: hand grip strength, BMI: body mass index, SMM: skeletal muscle mass, W: weight, H^2^: height square

While probable sarcopenia was associated with both ADL and IADL disability, SO (BMI- adjusted and W-adjusted) were not associated with either ADL or IADL disability. In terms of confirmed sarcopenia, confirmed sarcopenia only adjusted for BMI was associated with both ADL and IADL disability. Whereas confirmed sarcopenia adjusted for height^2^ suggested by the EWGSOP2 criteria was not associated with either ADL or IADL disability. Associations of operational definitions of sarcopenia with functional measures were shown at Table 4.

**Table 4:** Associations of operational definitions of sarcopenia with functional measures (univariate analyses)

	Impaired ADL	*p* value	Impaired IADL	*p* value
Probable sarcopenia				
No	74 (43.3%)	**0.03**	10 (5.8%)	**< 0.001**
Yes	129 (54.4%)		51 (21.5%)	
Confirmed sarcopenia/W				
No	158 (47.6%)	> 0.05	39 (11.7%)	**< 0.001**
Yes	45 (59.2%)		22 (28.9%)	
Confirmed sarcopenia/BMI				
No	132 (45.2%)	**0.004**	26 (8.9%)	**< 0.001**
Yes	71 (61.2%)		35 (30.2%)	
Confirmed sarcopenia/H^2^				
No	203 (49.9%)	> 0.05	61 (15%)	> 0.05
Yes	0 (0%)		0 (0%)	
Sarcopenic obesity/W				
No	188 (49.5%)	> 0.05	54 (14.2%)	> 0.05
Yes	15 (53.6%)		7 (25%)	
Sarcopenic obesity /BMI				
No	188 (49.5%)	> 0.05	54 (14.2%)	> 0.05
Yes	15 (53.6%)		7 (25%)	
Obesity				
No	119 (45.9%)	**0.04**	31 (12%)	**0.03**
Yes	84 (56.4%)		30 (20.1%)	

Categorical variables expressed as number (frequency)

ADL: activities of daily living, IADL: instrumental activities of daily living, BMI: body mass index, SMM: skeletal muscle mass, W: weight, H^2^: height square

**Fig. 2:**
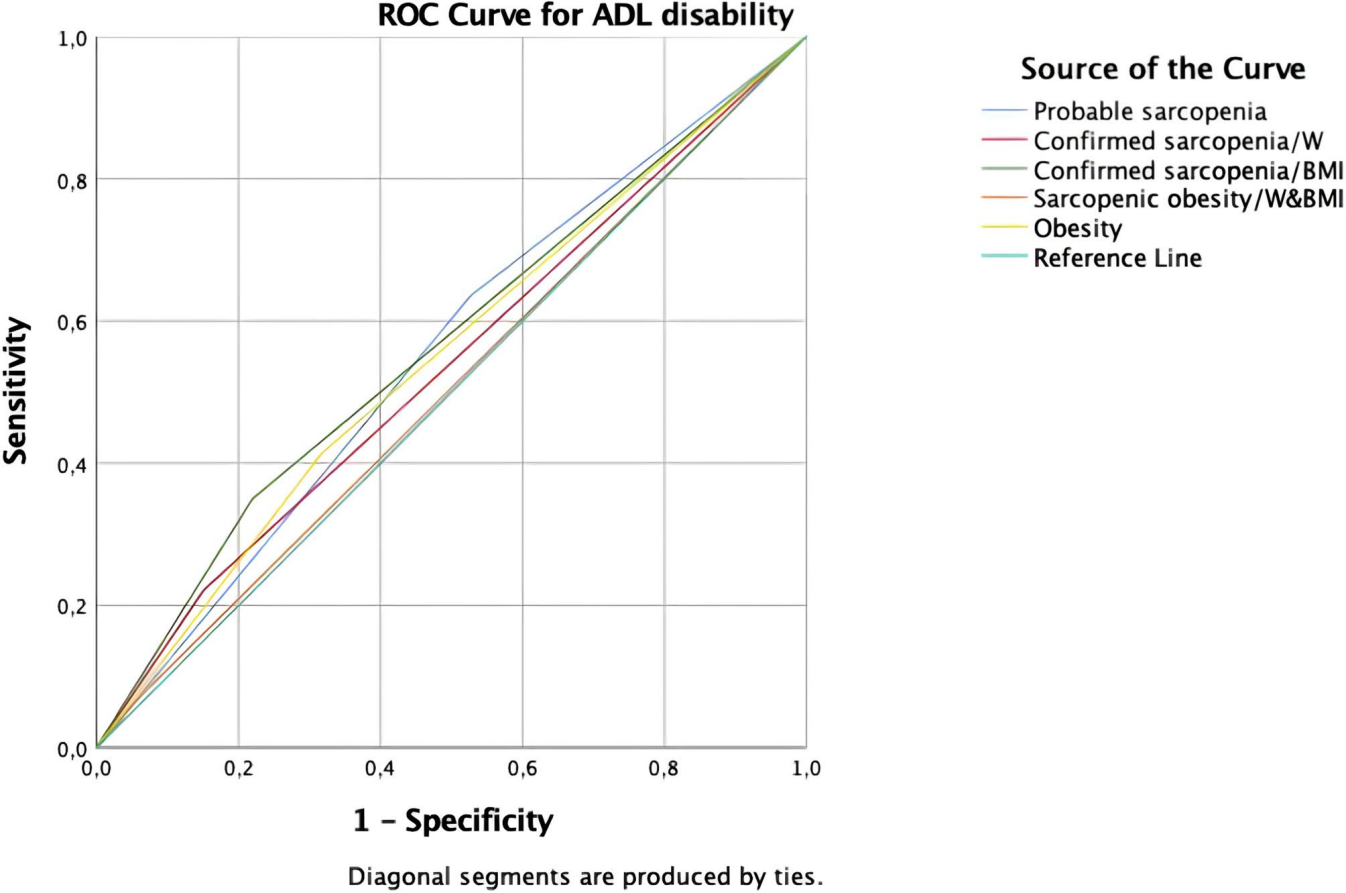
ROC curves of different operational definitons of sarcopenia and SO for predicting ADL disability. Individuals who had SO according to calculation adjusted by weight (6.9%) were the same patients according to calculation adjusted by BMI (6.9%). For this reason, it is shown with the same line on the ROC curve and expressed as ‘Sarkopenic obesity/W&BMI’

**Fig. 3:**
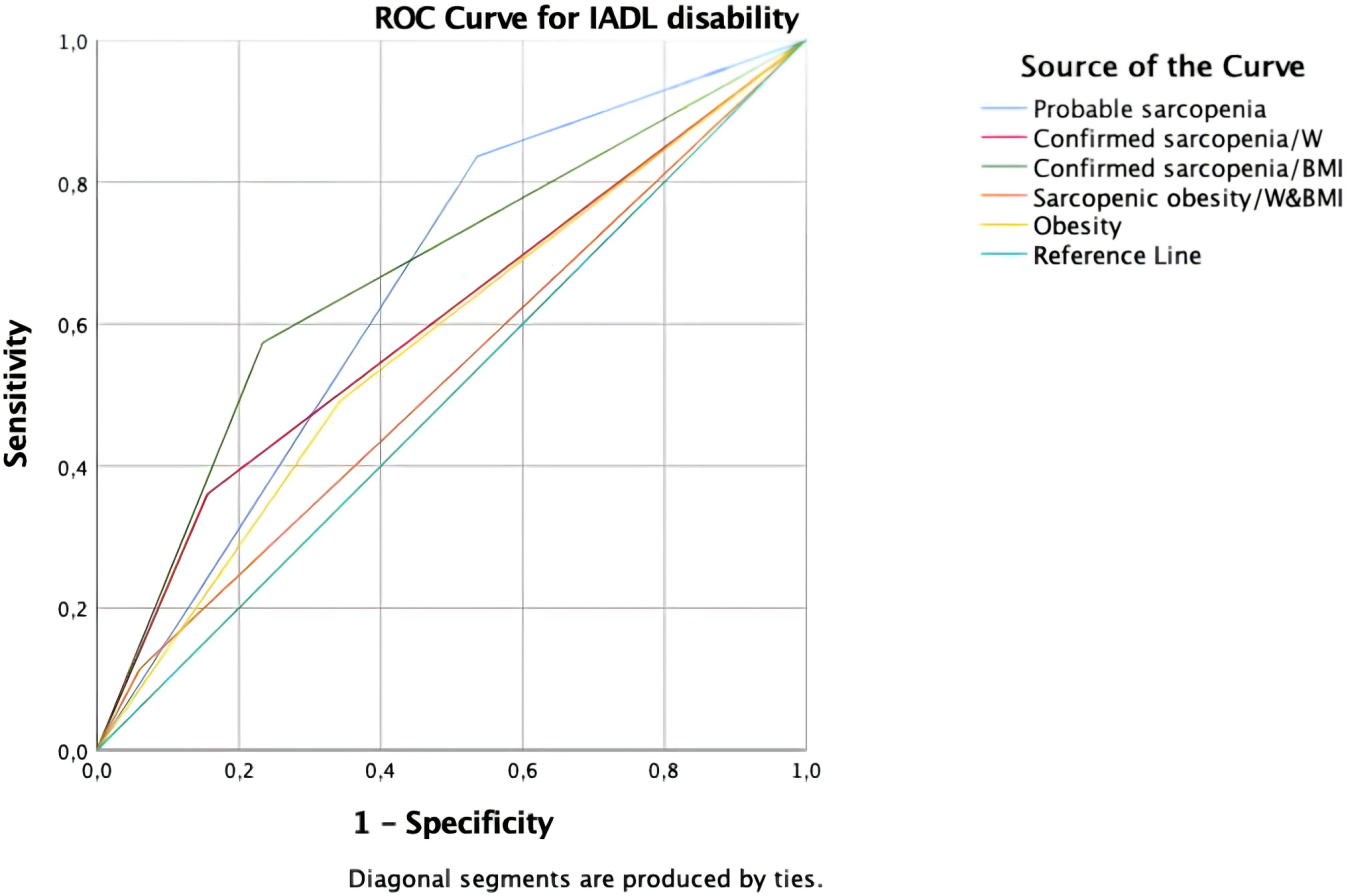
ROC curves of different operational definitons of sarcopenia and SO for predicitng IADL disability. Individuals who had SO according to calculation adjusted by weight (6.9%) were the same patients according to calculation adjusted by BMI (6.9%). For this reason, it is shown with the same line on the ROC curve and expressed as ‘Sarkopenic obesity/W&BMI’

Only confirmed sarcopenia by BMI reached significant p level while predicting ADLs in the ROC Curve. However this showed poor accuracy. ROC curves of different operational definitons of sarcopenia and SO for predicting ADL disability are shown in Fig. 2. The AUCs for the probable sarcopenia, confirmed sarcopenia (BMI-adjusted and W-adjusted) had fair accuracy for predicting IADL disability. ROC curves of different operational definitons of sarcopenia and SO for predicitng IADL disability are shown in Fig. 3. According to operational definitions of sarcopenia, probable sarcopenia had the highest sensitivity and NPV for predicting IADL disability. Receiver operating characteristic analysis for operational definitions of sarcopenia to predict impairment ADL and IADL were shown at Table 5.

**Table 5:** Receiver operating characteristic analysis for operational definitions of sarcopenia to predict impairment of ADL and IADL

	Impairment ADL					Impairment IADL				
	Sensitivity (%)	Specificity (%)	PPV (%)	NPV (%)	ROC AUC* (95% CI), significance (p)	Sensitivity (%)	Specificity (%)	PPV (%)	NPV (%)	ROC AUC* (95% CI), significance (p)
Probable sarcopenia	63.5	47.8	54.4	56.7	0.554 (0.499–0.610), 0.6	**83.6**	46.4	21.5	**94.2**	0.650 (0.582–0.718) **< 0.001**
Confirmed sarcopenia/W	22.2	84.9	59.2	52.4	0.535 (0.479–0.591) 0.22	36	84.4	29	88.3	0.603 (0.520–0.685) **0.01**
Confirmed sarcopenia/BMI	35	78.1	61.2	54.8	0.565 (0.509–0.621), **0.02**	**57.4**	76.7	30.2	**91.1**	0.670 (0.592–0.748) **< 0.001**
Confirmed sarcopenia/H^2^	0	99.5	0	50	0.498 (0.441–0.554) 0.932	0	99.7	0	85.1	0.499 (0.420–0.577) > 0.5
Sarcopenic obesity/W&BMI**	7.4	93.7	53.6	50.5	0.505 (0.449–0.561) 0.86	11.5	94	25	85.8	0.527 (0.446–0.608) 0.5
Obesity	41.4	68.3	56.4	54.1	0.548 (0.493–0.604) 0.09	49.2	65.7	20.1	88	0.574 (0.495–0.654) 0.06

*AUC indicates area under the ROC curve

** Individuals who had SO according to calculation adjusted by weight (6.9%) were the same patients according to calculation adjusted by BMI (6.9%). For this reason, it is shown with the same line on the ROC curve and expressed as ‘Sarcopenic obesity/W&BMI’

PPV: Positive predictive value, NPV: Negative predictive value, ADL: Activities of Daily Living, ROC: Receiver operating curve, AUC: Area under curve, BMI: body mass index, W: weight, H^2^: height square

## Discussion

Sarcopenic obesity is an increasing problem worldwide and it leads to significant health problems in older adults, such as the risk of developing comorbidities, geriatric syndromes [44], and functional limitation [45]. With the ESPEN/EASO consensus in 2022, the definition and diagnostic algorithm of SO were clarified providing a global algorithm to make it easier to find the target population and apply interventions in terms of preserving functionality [18]. However, there are still gaps on the associations of functionality and SO with the new algorithm, as well as the associations of functionality with different SMM adjustments for sarcopenia component of SO [31]. To our knowledge, there is no study investigating the predictivity of different operational definitions of sarcopenia and SO with ESPEN /EASO algorithm, using different SMM adjustments for functional disability. And also this is the first study using local thresholds in all the diagnostic steps of sarcopenia and SO with the new algorithm.

In our study, SO adjusted for W or BMI were not associated with either ADL or IADL disability and also did not predict functional disability. This result might be related with obesity paradox [45]. The median BMI in our patient population was 28 kg/m^2^, and the majority of patients were within the BMI range which obesity is protective. Though obesity is associated with both ADL and IADL impairment in our study it is known that the decreased risk of sarcopenia in older individuals with obesity “obesity paradox” was dependent on higher muscle mass and strength [45]. In Japanese older patients undergoing rehabilitation, SO by ESPEN/EASO evaluation was not associated with poor functional outcomes as well [26]. Moreover, SO by ESPEN/EASO was not statistically associated with ADL at acute discharge in patients with stroke [28]. Among older patients with sarcopenia, obesity might have a protective effect against the limitations of some functional measures, and physical performance [19–21]. On the other hand a recent systematic review and meta-analysis concluded that SO patients presented lower physical performance compared with sarcopenic nonobese patients [15]. In stroke patients admitted to a post-acute rehabilitation hospital, SO by ESPEN/EASO was negatively associated with improvements in ADL [24]. In asthma patients referred for comprehensive pulmonary rehabilitation, SO by ESPEN/EASO was associated with worse functional outcomes [25]. Scott et al. found that men aged 70 years and older with SO had an increased risk of ADL and IADL disability compared to those without sarcopenia or obesity [23]. Also, in the study by Schlussel et al., 1079 participants aged 65 years and older were evaluated with ESPEN/EASO consensus algorithm and SO was associated with Disability Score [22]. The prognostic significance of obesity in sarcopenic adults especially on functionality is still controversial [15].

In our study, probable sarcopenia and BMI-adjusted confirmed sarcopenia were associated with both ADL and IADL disability, W-adjusted confirmed sarcopenia was only associated with IADL disability. Probable sarcopenia is associated with worsening of functional disability such as ADL, and IADL in older population [46]. Sarcopenia is also related with functional disability in older adults as well [47]. In our study in terms of IADL the AUC values for the probable sarcopenia, and confirmed sarcopenia (BMI-adjusted and W-adjusted) had fair accuracy for predicting IADL disability. In a retrospective study published before ESPEN/EASO consensus, older adults over 60 years of age were evaluated for associations of SO versus sarcopenia alone with functionality [19]. In this study, it was shown that, when compared to the non-sarcopenic non-obese group, sarcopenia alone definitions whether probable or confirmed adjusted by BMI had more increased risks than SO definitions for impaired ADL, and for impaired IADL [19]. Although this study was not conducted with the ESPEN/EASO algorithm and different threshold values ​​were used, probable sarcopenia and BMI-adjusted confirmed sarcopenia had a risk for IADL dependency, similar with our study. However, in our study confirmed sarcopenia adjusted by BMI had poor accuracy for predicting ADL disability.

The other important issue to be discussed here is the SMM adjustments for sarcopenia. SMM adjusted for BMI was better associated with functionality, physical performance and frailty, in comparison with the adjustments made for height^2^ or weight [29]. In the study by Schluessel et al., ESPEN/ EASO consensus criteria was used and for sarcopenia component of SO, SMM adjustment by W was applied as suggested in consensus criteria and SO was associated with disability [22]. In a study which compares different SO definitions for predicting mortality in a prospective cohort with advanced non-small cell lung cancer, although SMM/BMI was better associated with survival than SMM/W, SO (SMM/BMI) did not show an advantage in predicting survival over SO (SMM/W) [48]. In our study we found that the adjustment for BMI had higher sensitivity and higher NPV than the adjustment for W for IADL disability.

In our study, according to operational definitions of sarcopenia, probable sarcopenia had the highest sensitivity and NPV for predicting IADL disability. Though muscle measures both muscle mass and strength are predictors of future ADLs and IADLs dependency in the older adult population [49], muscle weakness is reported to predict poor outcomes better than muscle mass [6, 21, 50]. 

## Conclusion

We found that probable sarcopenia (with the highest sensitivity and NPV) and confirmed sarcopenia adjusted by BMI (higher sensitivity and NPV than adjustment by W) and confirmed sarcopenia adjusted by W are better predictors of IADL disability than SO defined with ESPEN/EASO consensus algorithm. To fully understand the dual burden of sarcopenia and obesity, it is necessary to investigate the underlying mechanisms, in particular their impact on functional capacity. Also, future studies need to refine the definitions of sarcopenic obesity and investigate its distinct impact on functional impairment compared to sarcopenia alone.

### Limitation and strengths

To our knowledge, there is no study investigating the predictivity of different operational definitions of sarcopenia and SO with ESPEN /EASO algorithm, using different SMM adjustments for functional disability. And also, this is the first study using local thresholds in all the diagnostic steps of sarcopenia and SO with the new algorithm. However, there are important limitations. There might be a selection bias because this study is a single centered study conducted in a university hospital. The generalizability of the study is limited. Waist circumference is also recommended in the screening phase of obesity in addition to BMI. However due to the retrospective design waist circumference was not available in our data. So it might cause some cases of SO with normal BMI but high waist circumference were likely missed. Also, the retrospective design might cause some missing data in terms of comorbid diseases that were not recorded in patient files. Confounding variables such as nutritional status, and inflammation and cognitive status which may affect both patients’ independence in daily living activities and orientation in HGS measurements were not evaluated in the study.

Since the study is retrospective observational, it cannot provide a cause-and-effect relationship. For this, prospective longitudinal studies are needed.

## Data Availability

There are ethical restrictions on publicly sharing a de-identified dataset due to sensitive patient information. Data is available from the secretary of the Ege University Ethics Committee via email (egetaek@gmail.com) for researchers who meet the criteria for access to confidential data. The datasets used and/or analysed during the current study available from the corresponding author on reasonable request with permission of Ege University Ethics Committee.

## Abbreviations

S: Sarcopenia
SO: Sarcopenic obesity
ESPEN/EASO: European Society for Clinical Nutrition and Metabolism and the European Association for the Study of Obesity
EWGSOP2: Revised European Working Group on Sarcopenia in Older People
SMM: Skeletal muscle mass
W: Weight
BMI: Body mass index
H^2^: Height square
ADL: Activities of daily living
IADL: Instrumental ADL
ROC: Receiver operating characteristic
AUC: Area under ROC curve
HGS: Handgrip strength
BIA: Bioelectrical impedance analysis
PPV: Positive predictive value
NPV: Negative predictive value

## References

[1] Wang M, Tan Y, Shi Y, Wang X, Liao Z, Wei P. Diabetes and sarcopenic obesity: pathogenesis, diagnosis, and treatments. Front Endocrinol. 2020;11:568. 10.3389/fendo.2020.00568 PMC7477770 32982969

[2] Batsis JA, Villareal DT. Sarcopenic obesity in older adults: aetiology, epidemiology and treatment strategies. Nat Reviews Endocrinol. 2018;14(9):513–37. 10.1038/s41574-018-0062-9 PMC6241236 30065268

[3] Dowling L, Duseja A, Vilaca T, Walsh JS, Goljanek-Whysall K. MicroRNAs in obesity, sarcopenia, and commonalities for sarcopenic obesity: a systematic review. J cachexia Sarcopenia Muscle. 2022;13(1):68–85. 34984856 10.1002/jcsm.12878 PMC8818592

[4] Xie WQ, Xiao GL, Fan YB, He M, Lv S, Li YS. Sarcopenic obesity: research advances in pathogenesis and diagnostic criteria. Aging Clin Exp Res. 2021;33(2):247–52. 31845200 10.1007/s40520-019-01435-9

[5] Cruz-Jentoft AJ, Baeyens JP, Bauer JM, Boirie Y, Cederholm T, Landi F, et al. Sarcopenia: European consensus on definition and diagnosis: report of the European working group on sarcopenia in older people. Age Ageing. 2010;39(4):412–23. 20392703 10.1093/ageing/afq034 PMC2886201

[6] Cruz-Jentoft AJ, Bahat G, Bauer J, Boirie Y, Bruyère O, Cederholm T, et al. Sarcopenia: revised European consensus on definition and diagnosis. Age Ageing. 2019;48(1):16–31. 30312372 10.1093/ageing/afy169 PMC6322506

[7] Studenski SA, Peters KW, Alley DE, Cawthon PM, McLean RR, Harris TB et al. The FNIH sarcopenia project: rationale, study description, conference recommendations, and final estimates. The journals of gerontology Series A, Biological sciences and medical sciences. 2014;69(5):547– 58. 10.1093/gerona/glu010 PMC3991146 24737557

[8] Chen LK, Liu LK, Woo J, Assantachai P, Auyeung TW, Bahyah KS, et al. Sarcopenia in Asia: consensus report of the Asian working group for sarcopenia. J Am Med Dir Assoc. 2014;15(2):95–101. 24461239 10.1016/j.jamda.2013.11.025

[9] Chen LK, Woo J, Assantachai P, Auyeung TW, Chou MY, Iijima K, et al. Asian working group for sarcopenia: 2019 consensus update on sarcopenia diagnosis and treatment. J Am Med Dir Assoc. 2020;21(3):300–e72. 32033882 10.1016/j.jamda.2019.12.012

[10] Fielding RA, Vellas B, Evans WJ, Bhasin S, Morley JE, Newman AB, et al. Sarcopenia: an undiagnosed condition in older adults. Current consensus definition: prevalence, etiology, and consequences. International working group on sarcopenia. J Am Med Dir Assoc. 2011;12(4):249–56. 21527165 10.1016/j.jamda.2011.01.003 PMC3377163

[11] Diago-Galmés A, Guillamon-Escudero C, Tenías-Burillo JM, Soriano JM, Fernández-Garrido J. Sarcopenic obesity in Community-Dwelling Spanish adults older than 65 years. Nutrients. 2023;15(23). 10.3390/nu15234932 PMC10708010 38068790

[12] Koliaki C, Liatis S, Dalamaga M, Kokkinos A. Sarcopenic obesity: epidemiologic evidence, pathophysiology, and therapeutic perspectives. Curr Obes Rep. 2019;8(4):458–71. 31654335 10.1007/s13679-019-00359-9

[13] Bahat G, Kilic C, Topcu Y, Aydin K, Karan MA. Fat percentage cutoff values to define obesity and prevalence of sarcopenic obesity in community-dwelling older adults in Turkey. Aging Male: Official J Int Soc Study Aging Male. 2020;23(5):477–82. 10.1080/13685538.2018.1530208 30422757

[14] Donini LM, Busetto L, Bischoff SC, Cederholm T, Ballesteros-Pomar MD, Batsis JA, et al. Definition and diagnostic criteria for sarcopenic obesity: ESPEN and EASO consensus statement. Clinical nutrition (Edinburgh. Scotland). 2022;41(4):990–1000. 10.1016/j.clnu.2021.11.014 35227529

[15] Eitmann S, Matrai P, Hegyi P, Balasko M, Eross B, Dorogi K, et al. Obesity paradox in older sarcopenic adults - a delay in aging: A systematic review and meta-analysis. Ageing Res Rev. 2024;93:102164. 38103840 10.1016/j.arr.2023.102164

[16] Gandham A, Mesinovic J, Jansons P, Zengin A, Bonham MP, Ebeling PR, et al. Falls, fractures, and areal bone mineral density in older adults with sarcopenic obesity: A systematic review and meta-analysis. Obes Reviews: Official J Int Association Study Obes. 2021;22(5):e13187. 10.1111/obr.13187 33491333

[17] Öztürk ZA, Türkbeyler İH, Abiyev A, Kul S, Edizer B, Yakaryılmaz FD, et al. Health-related quality of life and fall risk associated with age-related body composition changes; sarcopenia, obesity and sarcopenic obesity. Intern Med J. 2018;48(8):973–81. 29665258 10.1111/imj.13935

[18] Donini LM, Busetto L, Bischoff SC, Cederholm T, Ballesteros-Pomar MD, Batsis JA, et al. Definition and diagnostic criteria for sarcopenic obesity: ESPEN and EASO consensus statement. Obes Facts. 2022;15(3):321–35. 35196654 10.1159/000521241 PMC9210010

[19] Bahat G, Kilic C, Ozkok S, Ozturk S, Karan MA. Associations of sarcopenic obesity versus sarcopenia alone with functionality. Clin Nutr. 2021;40(5):2851–9. 33940398 10.1016/j.clnu.2021.04.002

[20] Saito H, Matsue Y, Kamiya K, Kagiyama N, Maeda D, Endo Y, et al. Sarcopenic obesity is associated with impaired physical function and mortality in older patients with heart failure: insight from FRAGILE-HF. BMC Geriatr. 2022;22(1):556. 35787667 10.1186/s12877-022-03168-3 PMC9254413

[21] Ozkok S, Aydin CO, Sacar DE, Catikkas NM, Erdogan T, Bozkurt ME, et al. Sarcopenic obesity versus sarcopenia alone with the use of probable sarcopenia definition for sarcopenia: associations with frailty and physical performance. Clin Nutr. 2022;41(11):2509–16. 36219979 10.1016/j.clnu.2022.09.005

[22] Schluessel S, Huemer MT, Peters A, Drey M, Thorand B. Sarcopenic obesity using the ESPEN and EASO consensus statement criteria of 2022 - Results from the German KORA-Age study. Obes Res Clin Pract. 2023;17(4):349–52. 37633820 10.1016/j.orcp.2023.08.002

[23] Scott D, Blyth F, Naganathan V, Le Couteur DG, Handelsman DJ, Waite LM, et al. Sarcopenia prevalence and functional outcomes in older men with obesity: comparing the use of the EWGSOP2 sarcopenia versus ESPEN-EASO sarcopenic obesity consensus definitions. Clin Nutr. 2023;42(9):1610–8. 37481869 10.1016/j.clnu.2023.07.014

[24] Yoshimura Y, Wakabayashi H, Nagano F, Matsumoto A, Shimazu S, Shiraishi A, et al. The applicability of the ESPEN and EASO-Defined diagnostic criteria for sarcopenic obesity in Japanese patients after stroke: prevalence and association with outcomes. Nutrients. 2022;14:19. 10.3390/nu14194205 PMC9570818 36235857

[25] Meys R, Machado FVC, Spruit MA, Stoffels AAF, van Hees HWH, van den Borst B, et al. Frequency and functional consequences of low appendicular lean mass and sarcopenic obesity in patients with asthma referred for pulmonary rehabilitation. Obes Facts. 2023;16(5):435–46. 37232056 10.1159/000531196 PMC10601668

[26] Shimizu A, Maeda K, Ueshima J, Inoue T, Murotani K, Ohno T, et al. Prevalence of sarcopenic obesity based on newly proposed diagnostic criteria and functional outcomes in older adults undergoing rehabilitation. Mech Ageing Dev. 2022;208:111728. 36084796 10.1016/j.mad.2022.111728

[27] Demirdağ F, Kıvrak Güçer B, Kolbaşı EN. Sarcopenic obesity is not associated with sexual dysfunction in older adults: a cross-sectional study. Aging Male: Official J Int Soc Study Aging Male. 2023;26(1):2252502. 10.1080/13685538.2023.2252502 37905446

[28] Abe T, Yoshimura Y, Sato Y, Nagano F, Matsumoto A. Applicability of the diagnostic criteria for sarcopenic obesity defined by ESPEN/EASO criteria in acutely admitted patients with stroke: prevalence and association with outcomes. J Nutri Sci Vitaminol. 2023;69(6):454–62. 10.3177/jnsv.69.454 38171818

[29] Bahat G, Kilic C, Ilhan B, Karan MA, Cruz-Jentoft A. Association of different bioimpedanciometry estimations of muscle mass with functional measures. Geriatr Gerontol Int. 2019;19(7):593–7. 31006968 10.1111/ggi.13668

[30] Bahat G, Ozkok S. How to adjust muscle mass while defining sarcopenia component of sarcopenic obesity: is body weight sufficient enough to represent body size? Aging Clin Exp Res. 2023;35(3):723–4. 36622546 10.1007/s40520-022-02326-2

[31] Shimizu A, Inoue T, Maeda K. Impact of sarcopenic obesity on functional outcomes. Aging. 2023;15(4):882–3. 36842098 10.18632/aging.204549 PMC10008493

[32] Kinoshita K, Satake S, Matsui Y, Arai H. Association between sarcopenia and fall risk according to the muscle mass adjustment method in Japanese older outpatients. J Nutr Health Aging. 2021;25(6):762–6.34179931 10.1007/s12603-021-1620-8

[33] Janssen I, Heymsfield SB, Baumgartner RN, Ross R. Estimation of skeletal muscle mass by bioelectrical impedance analysis. J Appl Physiol (Bethesda Md: 1985). 2000;89(2):465–71. 10.1152/jappl.2000.89.2.465 10926627

[34] Gallagher D, Heymsfield SB, Heo M, Jebb SA, Murgatroyd PR, Sakamoto Y. Healthy percentage body fat ranges: an approach for developing guidelines based on body mass index. Am J Clin Nutr. 2000;72(3):694–701. 10966886 10.1093/ajcn/72.3.694

[35] Bahat G, Aydin CO, Tufan A, Karan MA, Cruz-Jentoft AJ. Muscle strength cutoff values calculated from the young reference population to evaluate sarcopenia in Turkish population. Aging Clin Exp Res. 2021;33(10):2879–82. 33501623 10.1007/s40520-021-01785-3

[36] Özgür Y, Sayaca NA, Subaşı CF, Keskin Ö. Review of the Cut-off thresholds for muscle masses in diagnosis of sarcopenia and creation of a new appendicular muscle mass Estimation equation suitable for the Turkish population. Clin Sci Nutr. 2023;4(3):98–106.

[37] Savas S, Kilavuz A, Kayhan Koçak F, Cavdar S. Comparison of grip strength measurements by widely used three dynamometers in outpatients aged 60 years and over. J Clin Med. 2023;12(13). 10.3390/jcm12134260 PMC10342845 37445293

[38] Ates Bulut E, Soysal P, Dokuzlar O, Kocyigit SE, Aydin AE, Yavuz I, et al. Validation of population-based cutoffs for low muscle mass and strength in a population of Turkish elderly adults. Aging Clin Exp Res. 2020;32(9):1749–55. 31898170 10.1007/s40520-019-01448-4

[39] Katz S. Assessing self-maintenance: activities of daily living, mobility, and instrumental activities of daily living. J Am Geriatr Soc. 1983;31(12):721–7. 6418786 10.1111/j.1532-5415.1983.tb03391.x

[40] Lawton MP, Moss M, Fulcomer M, Kleban MH. A research and service oriented multilevel assessment instrument. J Gerontol. 1982;37(1):91–9. 7053405 10.1093/geronj/37.1.91

[41] Akobeng AK. Understanding diagnostic tests 1: sensitivity, specificity and predictive values. Acta paediatrica (Oslo, Norway: 1992). 2007;96(3):338– 41. 10.1111/j.1651-2227.2006.00180.x 17407452

[42] Power L, Mullally D, Gibney ER, Clarke M, Visser M, Volkert D, et al. A review of the validity of malnutrition screening tools used in older adults in community and healthcare settings - A MaNuEL study. Clin Nutr ESPEN. 2018;24:1–13. 29576345 10.1016/j.clnesp.2018.02.005

[43] Kaluźniak-Szymanowska A, Krzymińska-Siemaszko R, Lewandowicz M, Deskur-Śmielecka E, Stachnik K, Wieczorowska-Tobis K. Diagnostic performance and accuracy of the MNA-SF against GLIM criteria in Community-Dwelling older adults from Poland. Nutrients. 2021;13(7). 10.3390/nu13072183 PMC8308417 34202898

[44] Prado CM, Batsis JA, Donini LM, Gonzalez MC, Siervo M. Sarcopenic obesity in older adults: a clinical overview. Nat Reviews Endocrinol. 2024;20(5):261–77. 10.1038/s41574-023-00943-z PMC12854800 38321142

[45] Liu C, Wong PY, Chung YL, Chow SK, Cheung WH, Law SW, et al. Deciphering the obesity paradox in the elderly: A systematic review and meta-analysis of sarcopenic obesity. Obes Reviews: Official J Int Association Study Obes. 2023;24(2):e13534. 10.1111/obr.13534 36443946

[46] Çavdar S, Kocak FOK, Savas S. The association of muscle weakness with functional disability in older patients with diabetes mellitus: measured by three different grip strength thresholds. PLoS ONE. 2025;20(1):e0317250. 39883612 10.1371/journal.pone.0317250 PMC11781639

[47] Champaiboon J, Petchlorlian A, Manasvanich BA, Ubonsutvanich N, Jitpugdee W, Kittiskulnam P, et al. Calf circumference as a screening tool for low skeletal muscle mass: Cut-off values in independent Thai older adults. BMC Geriatr. 2023;23(1):826. 38066438 10.1186/s12877-023-04543-4 PMC10709895

[48] Zhou J, Luo L, Xie L, Hu S, Tan L, Lei X, et al. Sarcopenic obesity by the ESPEN/EASO criteria for predicting mortality in advanced non-small cell lung cancer. Clin Nutr. 2023;42(6):817–24. 37084468 10.1016/j.clnu.2023.04.010

[49] Wang DXM, Yao J, Zirek Y, Reijnierse EM, Maier AB. Muscle mass, strength, and physical performance predicting activities of daily living: a meta-analysis. J cachexia Sarcopenia Muscle. 2020;11(1):3–25. 31788969 10.1002/jcsm.12502 PMC7015244

[50] Zhou W, Tong J, Wen Z, Mao M, Wei Y, Li X, et al. Prevalence and factors associated with dynapenia among middle-aged and elderly people in rural Southern China. Prev Med Rep. 2024;38:102630. 38375165 10.1016/j.pmedr.2024.102630 PMC10874841

